# Histone deacetylase inhibition up-regulates MHC class I to facilitate cytotoxic T lymphocyte-mediated tumor cell killing in glioma cells

**DOI:** 10.7150/jca.34471

**Published:** 2019-09-07

**Authors:** Ting Sun, Yanyan Li, Wei Yang, Haibin Wu, Xuetao Li, Yulun Huang, Youxin Zhou, Ziwei Du

**Affiliations:** 1 Neurosurgery & Brain and Nerve Research Laboratory, The First Affiliated Hospital of Soochow University, Suzhou, Jiangsu, China; 2 State Key Laboratory of Radiation Medicine and Protection, School of Radiation Medicine and Protection and Collaborative Innovation Center of Radiation Medicine of Jiangsu Higher Education Institutions, Soochow University, Suzhou, Jiangsu, China.

**Keywords:** Histone deacetylase inhibitors, Antigen presentation, Cytotoxic T lymphocytes, Immune recognition

## Abstract

**Background**: Immune cells recognize tumor antigens presented on major histocompatibility complex class I (MHC-I) molecule. Increase of MHC-I molecular expression makes tumor cells more susceptible to lysis by immune cells.

**Methods**: Tumor lysate vaccine was prepared to damage glioma cells including cell lines and primary cultured cells from surgical samples. The enhanced effect of histone deacetylase inhibitors (HDACi) to tumor lysate vaccine was observed. The expressions of MHC-I pathway molecules were detected by flow cytometry and western blot after HDACi treatment. Cell apoptosis and cell lysis were measured following blocking cytotoxic T lymphocyte (CTL) pathway. Tumor size and mice survival were analyzed in combinative treatment with HDACi and tumor lysate.

**Results**: HDACi up-regulated the expressions of MHC-I pathway molecules, and enhanced the recognition and killing of immune cells, which was activated by tumor lysate. Activated antigen specific immune responses regulated CTL activity, and HDACi promoted immune response through cytotoxic effect of CTL. Anti-tumor effect of tumor lysate pulse immunogenicity *in vivo* was elevated by HDACi due to up-regulation of antigen presentation.

**Conclusions**: Our study showed that HDACi enhanced recognition of glioma cell by immune cells and sensitivity of tumor immunotherapy, and improved the anti-tumor effect of tumor lysate vaccine through activating CTL immune response. These pharmacological molecular mechanisms of increasing immune recognition suggest that epigenetic modulation is a promising strategy for sensitizing immunotherapy for glioma treatment.

## Introduction

The immune system identifies and acts against tumor cells by adaptive cell reactions of CD8^+^ cytotoxic T lymphocytes (CTLs) and natural killer cells, which play a critical role in restraining tumor development. CTLs recognize tumor antigens presented on major histocompatibility complex class I (MHC-I) molecule and lyse cancer cells 1-3. Increase of the expression of MHC-I molecule can be of therapeutic significance since it makes tumor cells more susceptible to lysis by CTLs 4,5.

Presentation of tumor associated antigens by MHC-I molecule is regulated by the transcription of antigen processing machinery (APM) genes involving low molecular mass proteins LMP-2 and LMP-7 and transporters associated with antigen processing TAP-1 and TAP-2. Tumor associated antigens expressed by malignant cells are transported by TAP-1 and TAP-2 into the endoplasmic reticulum and loaded onto MHC-I, then translocate to the cell surface to present to CD8^+^ CTLs. Thus, cellular immunity can function as an effective tumor detection and elimination system 6,7.

There is a significant prognostic factor for overall survival in the presence of tumor infiltrating lymphocytes (TILs) in the tumor area 8. It is generally admitted that CD8^+^ T cells are directly involved in antitumor cytotoxic responses. Antitumor immunity function of CD8^+^ T cell was demonstrated using tumor specific CTLs from peripheral blood or tumor tissue of patients with diverse cancers. Immune modulatory functions of histone deacetylase inhibitors (HDACi) include modulation of regulatory T cells (Treg) and changes in TILs composition 9. Emerging data suggest that epigenetic modulation is important for controlling T cells infiltration and differentiation 10.

In this study, we demonstrated that glioma cells lysed by specific CTLs. Increased surface expressions of MHC-I and cellular TAPs and LMPs molecules on glioma cells were induced by HDACi, which led to enhanced presentation of tumor associated antigens. We validated the HDACi elicited immunogenicity of glioma and CTLs specific lysing efficacy.

## Materials and Methods

### Primary cell culture of human glioma and cell lines

Surgically removed glioma biopsies were obtained from the department of neurosurgery of the First Affiliated Hospital of Soochow University. The use of human tissues was approved by the ethics committee of the First Affiliated Hospital of Soochow University. Fresh glioma samples were washed and minced in phosphate buffered saline (PBS) followed by enzymatic dissociation at 37°C for 20 min. The isolated cells were cultured in high glucose Dulbecco's modified Eagle's medium (DMEM) with 10% fetal bovine serum (FBS). Human glioma cell line U251 and mouse glioma cell line GL261 were cultured in high glucose DMEM with 10% FBS. Cells were cultured at 37°C in a humidified 5% CO_2_ atmosphere.

### Glioma implantation mice model

The male syngenic C57BL/6J mice about 18-20 g were bred and housed in a specific pathogen free animal facility. All animal experimental protocols were approved by the Institutional Animal Care and Use Committee of Soochow University and complied with the code of ethics for animal experimentation. The 10^6^ GL261 cells were injected into the thigh of the right hind leg of the C57BL/6J mice subcutaneously (s.c.). The tumor bearing mice were sacrificed when their body weight was decreased.

### T cell isolation and culture

T cells of mice were separated from spleen and lymph nodes of tumor bearing mice. T cells of human were from peripheral blood mononuclear cells (PBMC) matching with glioma patients. T cells cocultured with U251 cells were from PBMC of healthy donors. Tumor lysate was obtained by making tumor cells frozen and melt 3 times repeatedly. Human T cells were isolated and resuspended at a final concentration of 10^6^/ml, then pulsed with prepared tumor lysate from 10^7^ tumor cells. The generated CTLs were used after 6 days culture. Cells were kept at 37°C, 5% CO_2_ in DMEM containing 4.5g/l glucose and supplemented with 10% FBS, 10 μM Hepes, 20 μM β-mercaptoethanol, 50 IU/ml of human recombinant IL-2.

### CTLs cytotoxicity assays

Glioma cells were pre-labelled with 0.5μM CellTracker Green CMFDA (ThermoFisher Scientific, MA, USA) for 1 hour and washed. Specific CTLs were pooled by whole glioma cell lysate, washed and resuspended in media supplemented with 20 U/mL IL-2. Target cells were plated in a 96 U-bottom well plate, and treated with 2 μM suberoyanilide hydroxamic acid (SAHA) for 24 h. Then target cells were cocultured with CTLs at an effector-to-target (E:T) ratio of 10:1 (10^5^ :10^4^ cells/well, final volume of 100 μl). The same volume of complete medium without activated CTLs was added in the blank control group. The read-out was performed after 24 h. Prior to the test, target cells were incubated for 2 h with 1 μM Concanamycin A (CMA), an inhibitor of Granzyme, and/or 10 μg/ml the blocking Fas:Fc (FFc), a Fas/FasL inhibitor. Cell proliferation was measured by Green CMFDA positive cell number, and CTL cytotoxicity was detected by 7-AAD staining using flow cytometry.

### Flow cytometry

Cells were trypsinized to a single cell suspension and stained with PE-conjugated antibodies against human or mouse MHC-1 (BD Bioscience), apoptosis detection kit (Biolegend) or 7-AAD for 15 min at 4°C. Samples were analyzed by flow cytometry using FACScan (BD Biosciences). Data were analyzed using FlowJo Software version 7.2 and presented by mean fluorescence intensities (MFI) or positive cell number.

### Western blot analysis

Cells were collected and lysed in RIPA buffer. The lysate was centrifuged and the supernatants retained. Protein concentrations of the lysates were determined by the BCA protein assay kit (Thermo Scientific). Equal amounts protein was separated by 10 % SDS-PAGE, then transferred to Immobilon PVDF membranes. Nonspecific-binding sites on the membrane were blocked in 5 % nonfat dry milk. The primary antibodies included anti-human and mouse TAP1 (Cell Signaling), TAP2 (invitrogen), LMP2 (invitrogen) and LMP7 (Cell Signaling). Appropriate peroxidase-conjugated secondary antibodies were used to bind specific primary antibodies. The signal was detected using an ECL western blotting detection kit.

### Anti-glioma effect *in vivo*

Mouse GL261 cells (10^6^ cells/mouse) were injected s.c. in the thigh of the right hind leg of mice. Body weight and tumor volume were measured twice per week. The measurement of tumor volume was terminated when the first mouse died in control group. Mice were randomly divided into 4 groups including control, SAHA, tumor lysate and SAHA plus tumor lysate groups. The mice of tumor lysate group were treated with tumor lysate at 3 days after GL261 cells inoculated. 10^7^ tumor cells were frozen and melt 3 times to produce tumor lysate. Tumor lysate of GL261 cells was collected and injected intradermally into mice. SAHA (100 mg/kg) was administrated i.p. at 10, 12, 14, 16 days after tumor cells were inoculated. Survival analysis was used to compare the differences of each group according to survival time.

### Statistical analysis

Statistical analyses were carried out using SPSS version 19.0 (SPSS Inc., Chicago, IL, USA), and data was statistically determined by one-way ANOVA. The significance level was considered at *P* < 0.05. Each *in vitro* experiment was repeated at least three times. Overall mouse survival was estimated via Kaplan-Meier survival curve, and compared between groups via stratified log-rank tests.

## Results

### SAHA enhanced cytotoxic effects of tumor specific CTLs

**Figure 1:**
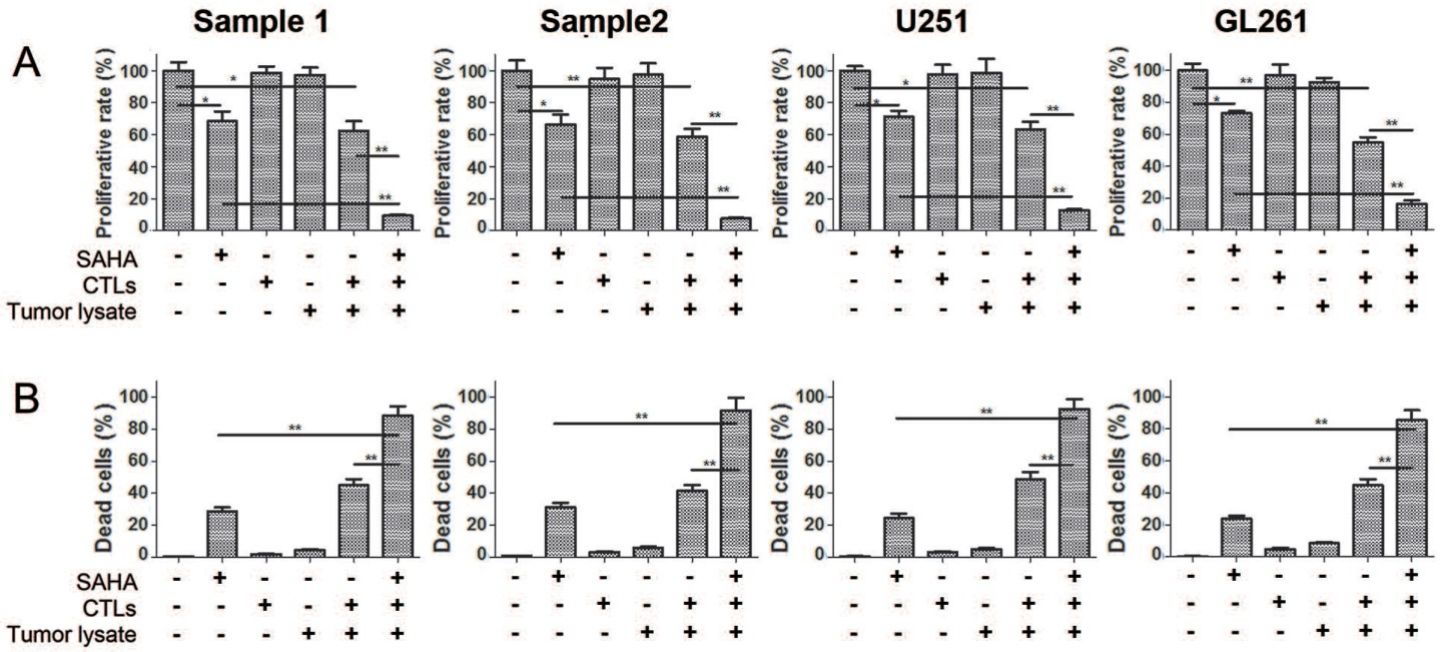
Elevated cytotoxic effects of SAHA, tumor lysate, and CTLs co-treatment for glioma cells Cell proliferation (A) and cell death (B) were measured using flow cytometry after cells were treated with 2 uM SAHA for 24 h, tumor lysate for 6 days or CTLs for 24 h. Proportions of proliferating normalised to controls are shown.*P < 0.05; **P < 0.01.

In order to observe killing effect of tumor specific CTLs on glioma cells, we assessed cytotoxicity on glioma cells after co-incubation with CTLs from spleen and lymph nodes of tumor bearing mice and PBMC of human in the presence of SAHA or not. Figure 1 showed that single CTLs or tumor lysate treatment had no effect on proliferation and cell death of glioma cells. Co-treatment of CTLs and tumor lysate decreased proliferation and cell death significantly comparing to CTLs treatment or tumor lysate alone. Co-treatment of SAHA, CTLs and tumor lysate strengthened the decrease of proliferation and cell death induced by CTLs and tumor lysate or SAHA treatment. These results demonstrate that specific CTLs from tumor lysate pulse have an effective anti-proliferation and cytotoxic function, and SAHA sensitizes glioma cells to specific CTLs-mediated cytotoxicity.

### SAHA up-regulated expressions of MHC-I, TAPs and LMPs

**Figure 2:**
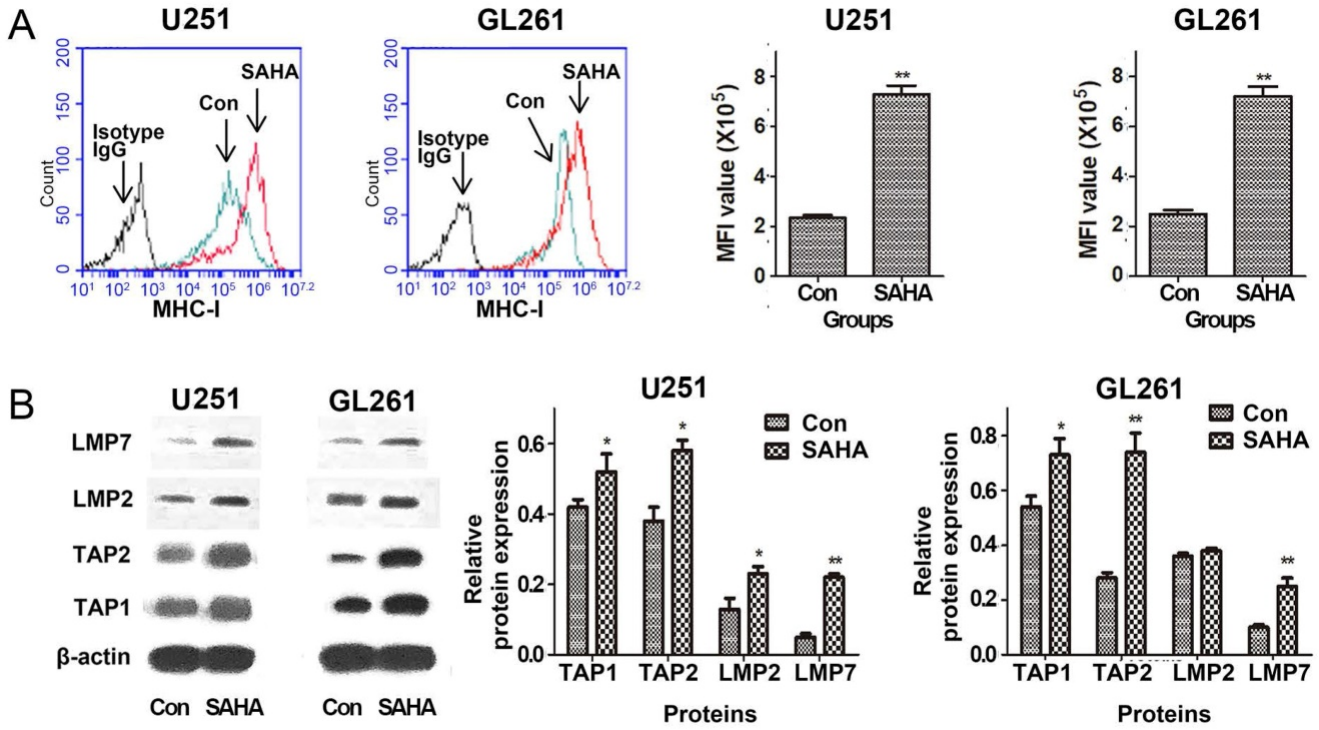
SAHA modulated the expression of antigen presenting molecules. Cells were treated with 2 uM SAHA for 24 h, and assessed for surface expression of MHC-I molecule using flow cytometry and TAPs and LMPs expressions using western blot. *P < 0.05; **P < 0.01.

MHC-I expression on surface of tumor cells may affect the ability of the CTLs to directly destroy tumor cells. The density of the peptide-MHC complexes at the surface can be affected by the efficiency of TAPs that shuttles peptides from cytosol into the endoplasmic reticulum and LMPs that generate peptides from proteins 11, 12. We explored the regulation of SAHA on constitutive expression of MHC-I, TAP1, TAP2, LMP2 and LMP7 proteins in glioma cells. The data of flow cytometry shown in Figure 2A demonstrated that SAHA up-regulated surface expression of MHC-I in U251 and GL261 cell lines. Expressions of TAP1, TAP2, LMP2 and LMP7 were analyzed by western blot. SAHA significantly up-regulated the 4 proteins expressions in U251 and GL261 cells except LMP2 in GL261 cells (Figure 2B). The results suggested that SAHA may increase the density of cell surface MHC-I and significantly up-regulated antigen presentation associated proteins expression.

### SAHA elevated glioma immune killing through CTL pathway

**Figure 3:**
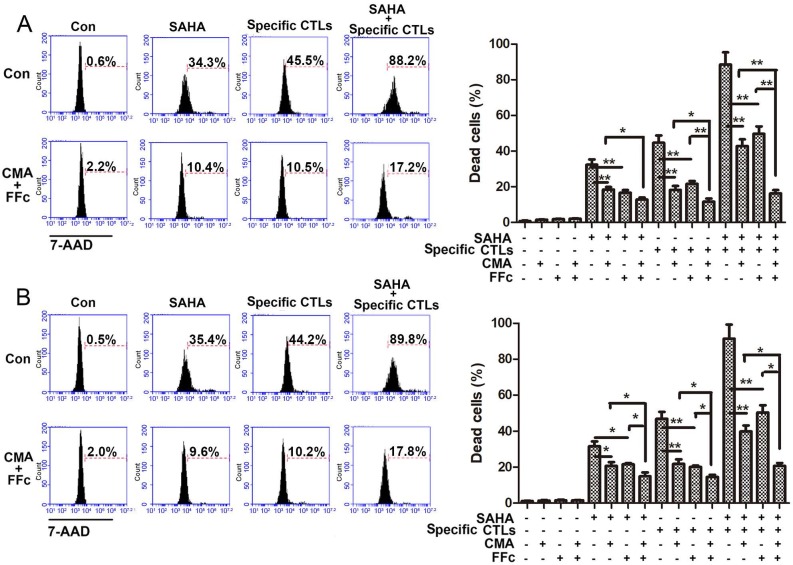
SAHA treatment increased CTL-mediated killing efficacy. Target cells U251 (A) or GL261 (B) were plated for 24 h with SAHA (2 μM) and labeled with CMFDA, then target cells were incubated with specific CTLs for 24 h. The effector: target (E:T) ratio used was 10:1. Cytotoxic effects of SAHA and CTLs were assayed by flow cytometry, and dead cells were stained by 7-AAD. CMA or FFc were pre-treated for 1 h to inhibit the CTL Perforin/Granzyme B or Fas/FasL pathway before SAHA and specific CTLs treatment. *P < 0.05; **P < 0.01.

In order to observe the mechanism of SAHA elevating tumor immune mechanism, whether cytotoxicity of SAHA for glioma cells was mediated by CTLs was observed. CMA was used to inhibit the CTL Perforin/Granzyme B pathway, and FFc fusion protein was used to inhibit the CTL Fas/FasL pathway. The inhibitory effects were detected after CTLs function 24 h with or without inhibitors pre-treatment. As shown in Figure 3, in U251 cells, co-treatment of CMA and FFc decreased cytotoxicity of SAHA and specific CTLs from 32.4±2.9% to 12.8±1.1% and from 44.6±4.1% to 11.5±1.8%, respectively. Treatment of specific CTLs with SAHA induced 88.5±6.9% cytotoxicity, CMA and FFc decreased cytotoxicity to 42.8±3.7% and 49.6±4.2 6%, respectively, and co-treatment of CMA and FFc decreased cytotoxicity to 16.1±1.9%. In CTLs plus tumor lysate pulse group, a partial inhibition of the CTL-mediated killing was observed when cells were pretreated with CMA or FFc. CMA and FFc inhibited 72.4% cytotoxicity when cells were co-treated with SAHA and specific CTLs, and 19.6% cytotoxicity was from SAHA. Cytotoxicity of specific CTLs decreased 33.1% after CMA and FFc inhibition. Consistent results were also showed in GL261 cells.

**Figure 4:**
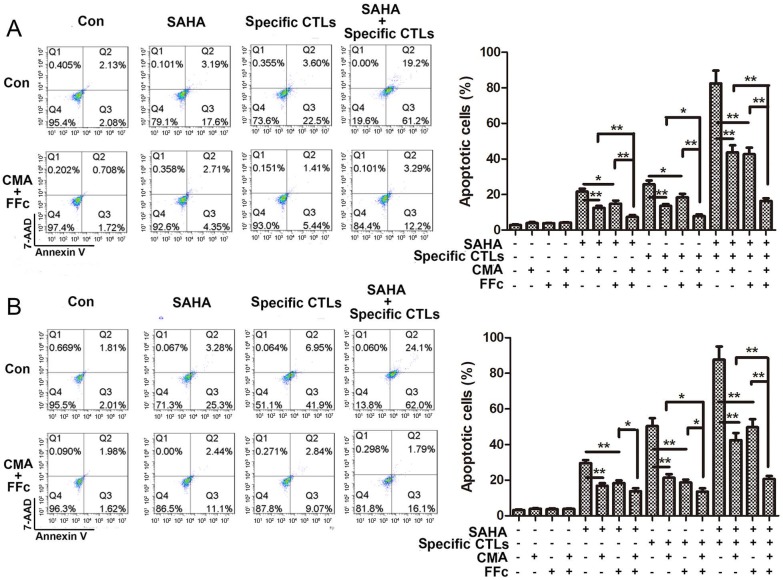
SAHA treatment enhanced CTL-mediated apoptosis in glioma cells. Glioma cells U251 (A) and GL261 (B) were treated with SAHA for 24 h, then incubated with specific CTLs for 12 h. Cells were pre-treated with CMA or FFc for 1 h to inhibit the CTL Perforin/Granzyme B or Fas/FasL pathway before SAHA and specific CTLs treatment. Cells were stained by Annexin V and 7-AAD for apoptotic assay. *P < 0.05; **P < 0.01.

Apoptosis of target cells stained by Annexin V and 7-AAD was detected using flow cytometry. The data in Figure 4 showed that combined treatment with SAHA and specific CTLs in U251 cells induced apoptosis of 82.5±7.2%, which was significant higher comparing with SAHA (21.6±1.7%) or specific CTLs (25.7±2.2%) treatment alone. Combination of inhibitor CMA and FFc decreased apoptosis to 16.4±1.5% when SAHA and specific CTLs were co-treated. We got the consistent results in GL261 cells. These data suggested that SAHA increased the effect of specific CTLs killing target cells, which can be reversed by CTL pathway inhibitors. Cytotoxicity and apoptotic ratio of glioma cells were significantly elevated due to enhancement of CTLs effect induced by SAHA through CTL pathway.

### Therapeutic efficacy *in vivo*

**Figure 5:**
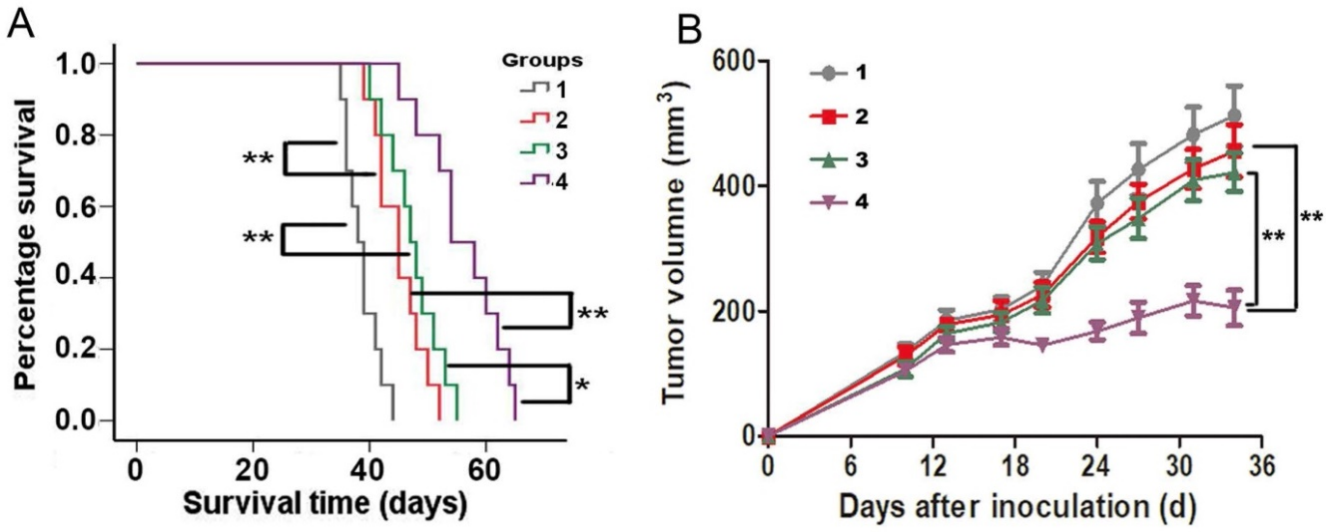
The efficacy of SAHA enhancing specific CTLs of tumor lysate pulse for killing glioma cells in C57BL/6 mice bearing GL261 cells allografts. Animals were treated with normal saline i.p. (group 1); administered SAHA i.p. (group 2); intradermally administered with tumor lysates (group 3) or administered with SAHA plus tumor lysates (group 4). (A) A longer period of survival was shown in mice that exhibited SAHA plus tumor lysates compared with controls, SAHA or tumor lysates alone. (B) Tumor size were monitored twice per week. Mean tumor volumes of each group ± SEM and *P* value for comparison between groups were showed. *P < 0.05, **P < 0.01

The efficacy of specific CTLs killing antigen-elevated glioma cells was evaluated in the GL261 subcutaneous allograft models. The status of the mice and suppression of tumor growth were monitored. Figure 5A showed survival results using Kaplan-Meier curve. Significant anti-tumor efficacy was observed in all treated groups. The mean survival times of the mice administered SAHA and tumor lysate pulse groups were 45.1 ± 4.2 days (95% CI, 42.5 - 47.7 days) and 45.1 ± 4.7 days (95% CI, 42.5 - 47.7 days), respectively, which were significantly increased compared with that of the control group (38.7 ± 3.2 days, 95% CI, 36.9 - 40.5 days, *P* < 0.01). Moreover, mean survival time of mice administered with SAHA plus tumor lysate pulse was 56.2 ± 6.7 days (95% CI, 51.3 - 61.0 days), which showed markedly statistical difference on comparing SAHA (*P* < 0.01) or tumor lysate pulse group (*P* < 0.05). The median survival times of control, SAHA, tumor lysate pulse and SAHA plus tumor lysate pulse groups were 38, 45, 47 and 54 days, respectively. The results indicated SAHA enhanced anti-tumor efficacy induced by tumor lysate specific activated CTLs.

Tumor growth was observed in Figure 5B when C57BL/6 mice bearing syngeneic mouse glioma GL261 tumor were administered SAHA and tumor lysate pulse. Combination of SAHA and tumor lysate pulse group was found to be more effective in inhibiting tumor growth *in vivo* at 34 days than SAHA or tumor lysate pulse group alone. These data were consistent with the results *in vitro*, which demonstrated that SAHA enhanced activated CTLs cytotoxicity of tumor lysate pulse.

## Discussion

Previous reports have shown that histone deacetylation was relative to the repression of MHC-I molecule in various mouse and human tumor cells 12-13. In addition, it has been found that HDACi modulate immune response, alter T cells activity and regulate cytokine expression 9,10,14. We detected the effect of SAHA, an efficient pan-class I and class II HDACi 15, enhancing tumor lysate specific CTLs to kill glioma cells. Our data demonstrated that SAHA up-regulated the expression of antigen presenting associated molecules, thus the immune response was enhanced through promoting tumor recognition by specific CTLs.

Our data showed that SAHA up-regulated the expression of MHC-I pathway components in mouse and human glioma cells. The data presented here are also consistent with previous observations that mRNA and surface expression of MHC-I in were enhanced when tumor cells were treated with HDACi, and the expression of these genes was regulated mainly at the transcriptional level. Treatment with HDACi enhances the expression of molecules (TAP1, TAP2, LMP2, LMP7, Tapasin and MHC-I) involved in antigen processing and presentation via the MHC-I pathway 16-18. MHC-I pathway components, which are responsible for the generation and transport of MHC-I complexes, are regulated by histone deacetylation in tumor cells. The mechanism for down-regulation of MHC-I transcription in tumor cells was chromatin repression mediated by HDAC 13. Thus, Elevation of MHC-I pathway components using epigenetic methods in tumor cells could potentially be an effective means to activate anti-tumor CTLs immunity.

In order to explore the mechanism of HDACi upregulating MHC class I pathway to enhance tumor immunity, CTL pathway was analyzed on anti-tumor effect of HDACi. Our data showed that CTLs play an important role in the process of SAHA anti-tumor immunity. Blockade of CTL pathway inhibited the cytotoxic effect of SAHA. When they are activated, antigen specific immune responses regulate cytotoxic T cells activity and help to avoid autoimmunity and maintain immune system homeostasis 3. In the tumor microenvironment, cancer cells suppress immune response and inhibit cytotoxic T cells activity. Our study found SAHA damaged immune suppress produced by cancer and activated CTLs activity because of elevation of MHC-I pathway.

Our *in vivo* data showed that whole tumor lysate pulse combined with SAHA decreased overall tumor growth and prolonged survival, which suggested that the therapeutic benefit is a consequence of SAHA-induced increase in tumor cells immunogenicity through antigen presenting molecule up-regulation, which led to increase of T cells recognition, function and activation. Consistent with our data, previous data also demonstrated that MHC expression was associated with therapeutic response to immunotherapy, better progression free survival, overall survival and CD4^+^ and CD8^+^ T cells tumor infiltrate in node-negative breast cancer 19, colorectal cancer 20 and melanoma 21. HDAC inhibition leaded to PD-L1 upregulation, increased HLA-DR tumor cells expression *in vitro*, and increased T cells tumor infiltration, longer survival and tumor growth inhibition *in vivo* 22.

Overall, we demonstrated that SAHA elevated the effect of tumor lysate vaccine and antigen presenting and processing phenotype of glioma cells. The anti-tumor effect was performed by specific CTLs. HDACi alters the MHC-I expression and elicits CTLs recognition for glioma cells, and therefore could serve as targets for existing and novel immunotherapies. Consequently, the use of SAHA for glioma therapy will be a promising immunosensitizing strategy that is potential in the context of tumor sensitization to T cell immunotherapy.

## Abbreviations

MHC-I: major histocompatibility complex class I
APM: antigen processing machinery
HDACi: histone deacetylase inhibitors
CTL: cytotoxic T lymphocyte
TILs: tumor infiltrating lymphocytes
PBMC: peripheral blood mononuclear cells
SAHA: suberoyanilide hydroxamic acid
